# Global Population Structure and Evolution of *Bordetella pertussis* and Their Relationship with Vaccination

**DOI:** 10.1128/mBio.01074-14

**Published:** 2014-04-22

**Authors:** Marieke J. Bart, Simon R. Harris, Abdolreza Advani, Yoshichika Arakawa, Daniela Bottero, Valérie Bouchez, Pamela K. Cassiday, Chuen-Sheue Chiang, Tine Dalby, Norman K. Fry, María Emilia Gaillard, Marjolein van Gent, Nicole Guiso, Hans O. Hallander, Eric T. Harvill, Qiushui He, Han G. J. van der Heide, Kees Heuvelman, Daniela F. Hozbor, Kazunari Kamachi, Gennady I. Karataev, Ruiting Lan, Anna Lutyńska, Ram P. Maharjan, Jussi Mertsola, Tatsuo Miyamura, Sophie Octavia, Andrew Preston, Michael A. Quail, Vitali Sintchenko, Paola Stefanelli, M. Lucia Tondella, Raymond S. W. Tsang, Yinghua Xu, Shu-Man Yao, Shumin Zhang, Julian Parkhill, Frits R. Mooi

**Affiliations:** ^a^ Centre for Infectious Diseases Research, Diagnostics and Screening (IDS), Centre for Infectious Diseases Control (CIb), National Institute of Public Health and the Environment (RIVM), Bilthoven, The Netherlands; ^b^ UMC St. Radboud Hospital, Nijmegen, The Netherlands; ^c^ Wellcome Trust Sanger Institute, Wellcome Trust Genome Campus, Hinxton, Cambridge, United Kingdom; ^d^ Swedish Institute for Communicable Disease Control (SMI), Solna, Sweden; ^e^ National Institute of Infectious Diseases, Shinjuku-ku, Tokyo, Japan; ^f^ Laboratorio VacSal, Instituto de Biotecnología y Biología Molecular, Facultad de Ciencias Exactas, Universidad Nacional de La Plata, CONICET, La Plata, Argentina; ^g^ Institut Pasteur, Molecular Prevention and Therapy of Human Infections, Paris, France; ^h^ Centre National de la Recherche Scientifique, URA-CNRS 30-12, Paris, France; ^i^ National Center for Immunization and Respiratory Diseases (NCIRD), Centers for Disease Control and Prevention (CDC), Atlanta, Georgia, USA; ^j^ Centers for Disease Control, Taipei, Taiwan, Republic of China; ^k^ Microbiology & Infection Control, Statens Serum Institut, Copenhagen, Denmark; ^l^ Public Health England—Respiratory and Vaccine Preventable Bacteria Reference Unit, Colindale, United Kingdom; ^m^ Department of Veterinary and Biomedical Sciences, The Pennsylvania State University, University Park, Pennsylvania, USA; ^n^ Department of Infectious Disease Surveillance and Control, National Institute for Health and Welfare, Finland; ^o^ Gamaleya Research Institute for Epidemiology and Microbiology, Ministry of Health Russian Federation, Moscow, Russian Federation; ^p^ School of Biotechnology and Biomolecular Sciences, University of New South Wales, Sydney, Australia; ^q^ National Institute of Public Health, National Institute of Hygiene, Warsaw, Poland; ^r^ Department of Pediatrics, Turku University Hospital, Turku, Finland; ^s^ Department of Biology and Biochemistry, University of Bath, Bath, United Kingdom; ^t^ Centre for Infectious Diseases and Microbiology—Public Health, Institute of Clinical Pathology and Medical Research, Westmead Hospital, Westmead, New South Wales, Australia; ^u^ Sydney Emerging Infectious Diseases and Biosecurity Institute, The University of Sydney, Sydney, New South Wales, Australia; ^v^ Department of Infectious, Parasitic & Immune-Mediated Diseases, Istituto Superiore di Sanita, Rome, Italy; ^w^ Laboratory for Syphilis Diagnostics and Vaccine Preventable Bacterial Diseases, National Microbiology Laboratory, Public Health Agency of Canada, Winnipeg, Manitoba, Canada; ^x^ National Institute for Food and Drug Control, Beijing, Republic of China

## Abstract

*Bordetella pertussis* causes pertussis, a respiratory disease that is most severe for infants. Vaccination was introduced in the 1950s, and in recent years, a resurgence of disease was observed worldwide, with significant mortality in infants. Possible causes for this include the switch from whole-cell vaccines (WCVs) to less effective acellular vaccines (ACVs), waning immunity, and pathogen adaptation. Pathogen adaptation is suggested by antigenic divergence between vaccine strains and circulating strains and by the emergence of strains with increased pertussis toxin production. We applied comparative genomics to a worldwide collection of 343 *B. pertussis* strains isolated between 1920 and 2010. The global phylogeny showed two deep branches; the largest of these contained 98% of all strains, and its expansion correlated temporally with the first descriptions of pertussis outbreaks in Europe in the 16th century. We found little evidence of recent geographical clustering of the strains within this lineage, suggesting rapid strain flow between countries. We observed that changes in genes encoding proteins implicated in protective immunity that are included in ACVs occurred after the introduction of WCVs but before the switch to ACVs. Furthermore, our analyses consistently suggested that virulence-associated genes and genes coding for surface-exposed proteins were involved in adaptation. However, many of the putative adaptive loci identified have a physiological role, and further studies of these loci may reveal less obvious ways in which *B. pertussis* and the host interact. This work provides insight into ways in which pathogens may adapt to vaccination and suggests ways to improve pertussis vaccines.

## IMPORTANCE

Whooping cough is mainly caused by *Bordetella pertussis*, and current vaccines are targeted against this organism. Recently, there have been increasing outbreaks of whooping cough, even where vaccine coverage is high. Analysis of the genomes of 343 *B. pertussis* isolates from around the world over the last 100 years suggests that the organism has emerged within the last 500 years, consistent with historical records. We show that global transmission of new strains is very rapid and that the worldwide population of *B. pertussis* is evolving in response to vaccine introduction, potentially enabling vaccine escape.

## INTRODUCTION

*Bordetella pertussis* is the primary causative agent of pertussis (whooping cough), a respiratory disease which is particularly severe for unvaccinated infants. Indeed, pertussis was a major cause of infant deaths before the introduction of vaccination. Even today, pertussis is a significant cause of child mortality, and estimates from the WHO suggest that, in 2008, about 16 million cases of pertussis occurred worldwide, 95% of which were in developing countries, and that about 195,000 children died from this disease (1).

There has been much speculation about the origin of pertussis. Although the disease has very characteristic symptoms and high mortality in unvaccinated children, references to pertussislike symptoms have not been found in the ancient European literature. The first documented pertussis epidemic occurred in Paris in 1578 (2). In the 16th and 17th centuries, descriptions of pertussis epidemics in Europe were documented more frequently in the literature, possibly suggesting an expansion of the disease (3). The apparent emergence of pertussis in Europe over the last 600 years may be due to import, as symptoms similar to pertussis were described in a classical Korean medical textbook from the 15th century (4).

The introduction of vaccination has significantly reduced the pertussis burden; however, in the 1990s, a resurgence of pertussis was observed in many highly vaccinated populations (5). The years 2010 to 2012 have seen particularly large outbreaks in Australia, the Netherlands, the United Kingdom, and the United States, with significant mortality in infants (6–10). The possible causes for the pertussis resurgence are still under debate and include waning vaccine-induced immunity, the switch from whole-cell vaccines (WCVs) to less effective acellular vaccines (ACVs), and pathogen adaptation (5, 11–13). The contributions of these causes probably differ from country to country. The importance of pathogen adaptation is suggested by the antigenic divergence of circulating strains from vaccine strains and the emergence of strains which produce more toxin (reviewed in reference 5). Antigenic divergence initially involved relatively few mutations, affecting up to 12 amino acids in the five *B. pertussis* proteins included in ACVs, i.e., filamentous hemagglutinin (FHA), pertactin (Prn), the Ptx A subunit (PtxA), serotype 2 fimbriae (Fim2), and serotype 3 fimbriae (Fim3). In the 1980s, strains emerged with a novel allele for the Ptx promoter, designated *ptxP3*. Strains carrying the *ptxP3* allele have been shown to produce more Ptx *in vitro* (14). Significantly, mutations in these six loci have been associated with clonal sweeps (15). The emergence of the *ptxP3* lineage is particularly remarkable because *ptxP3* strains have risen to predominance, replacing the resident *ptxP1* strains in many European countries, the United States, and Australia (14, 16–21). Furthermore, the emergence of *ptxP3* strains is associated with increases in pertussis notifications in at least two countries (14, 20). However, another study found that the resurgence of pertussis in the United States was correlated with the *fim3-2* allele and not with *ptxP3* (22). More recently, strains have emerged that do not express one or more components of pertussis vaccines, in particular, Prn and FHA (17, 23–25).

Together with at least 425 other genes, the genes for the five *B. pertussis* proteins used in ACVs belong to the so-called *Bordetella* virulence gene (Bvg) regulon, consisting of a sensory transduction system which translates environmental cues into changes in gene expression (26, 27). Low temperatures and high sulfate and nicotinic acid concentrations are signals known to suppress genes in the Bvg regulon (28). As essentially all known virulence-associated proteins require Bvg for their expression, Bvg activation is used to identify genes that play a role in the interaction with the host, even if the function of that gene is not known.

Key questions concerning pertussis are the origin of the disease, the forces that have driven the shifts in *B. pertussis* populations, and the role of these shifts in the resurgence of pertussis. To address these questions, we have determined the global population structure of *B. pertussis* by whole-genome sequencing of 343 strains from 19 countries isolated between 1920 and 2010. Phylogenetic analysis revealed a deep divergence between two lineages of *B. pertussis*, possibly suggesting two independent introductions of the organism from an unknown reservoir. Bayesian methods showed that the date of the common ancestor of one of these lineages correlates with the first descriptions of pertussis in Europe and that this lineage has increased in diversity subsequent to the introduction of vaccination. Our analyses revealed that many (putative) adaptive mutations occurred in the period in which the WCV was used, suggesting that vaccination was the major force driving changes in *B. pertussis* populations. Furthermore, we extend our previous observation that the mutation leading to the *ptxP3* allele occurred once and that the *ptxP3* strains have spread and diversified worldwide (29). Finally, we identified novel putative adaptive loci, the analysis of which may cast new light on the persistence and resurgence of pertussis and point to ways to increase the effectiveness of vaccination.

## RESULTS AND DISCUSSION

### Phylogeny and phylogeography of *B. pertussis*.

**FIG 1:**
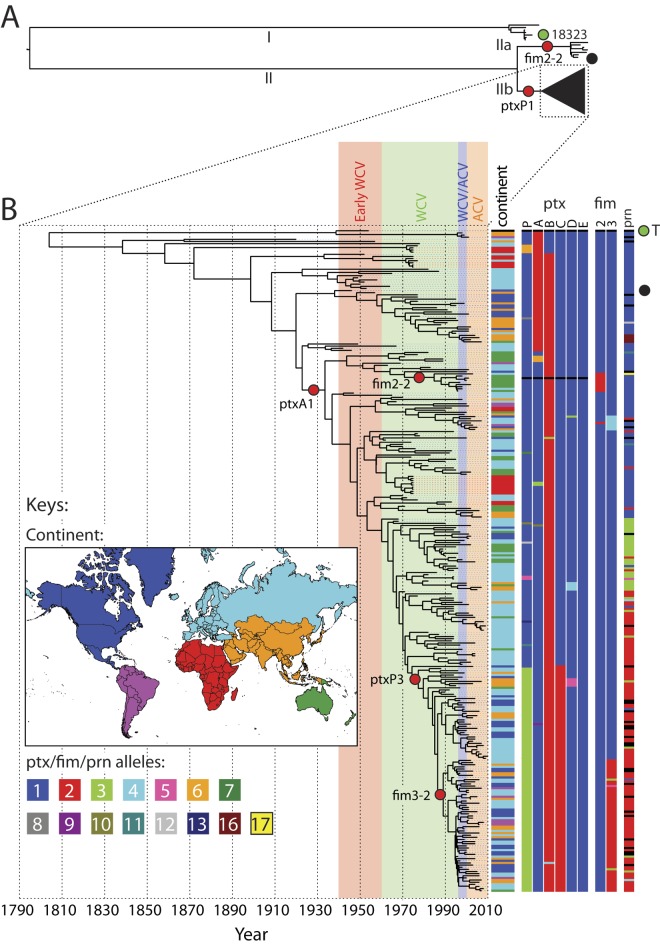
Global phylogeny of *B. pertussis*. (A) Outline of the maximum likelihood phylogeny of all *B. pertussis* samples sequenced, showing the deep divergence between lineages I and II. The complete tree is shown in Fig. S1 in the supplemental material. (B) Bayesian phylogeny of samples for which date information was available within the most common clade of *B. pertussis*. The position of a node along the *x* axis of the tree represents the median date reconstructed for that node across all sampled trees. Dates of whole-cell vaccine (WCV) and acellular vaccine (ACV) periods are shown as background colors behind the tree. To the right of the tree, the continent of origin of isolates is indicated by the first column of horizontal bars, colored according to the inset key. The remaining nine columns represent loci within the *ptx* operon, the *fim2* and *fim3* loci, and the *prn* locus, with assigned numerical alleles colored according to the key. The positions of reference strains 18323 and Tohama I (T) are indicated in panels A and B with green filled circles. Black filled circles represent the American vaccine strains B308 (A) and B310 (B) (Table S1). Red circles indicate the major changes in antigen gene alleles in proteins used in current ACVs (from *ptxA2* to *ptxA1*, *fim2-1* to *fim2-2*, *ptxP1* to *ptxP3*, and *fim3-1* to *fim3-2*).

We explored the evolutionary relationships among 343 *B. pertussis* strains collected from 19 countries representing six continents. Strains were isolated between 1920 and 2010 (Table 1; see Table S1 in the supplemental material). Illumina reads were aligned to the reference genome *B. pertussis* Tohama I (30), and 5,414 single-nucleotide polymorphisms (SNPs) were identified (Table S2), corresponding to a mean SNP density of 0.0013 SNPs/bp and an estimated mutation rate of 2.24 × 10^−7^ per site per year. We generated a maximum likelihood phylogeny representing the *B. pertussis* global population structure (Fig. 1A; Fig. S1). This phylogeny revealed two deep branches separated by 1,711 SNPs. Branch I contained only a small number of strains (1.7%), which were isolated between 1954 and 2000 and harbor *ptxA5* and *ptxP4* alleles (coding for the Ptx A subunit and the Ptx promoter, respectively), which are infrequently isolated nowadays. This branch includes the type strain 18323. Branch II contained strains isolated between 1920 and 2010 which fall into the more common *ptxA2 ptxP1*, *ptxA1 ptxP1*, and *ptxA1 ptxP3* types (Fig. 1B). Bayesian phylogenetic analysis estimated that these two lineages diverged around 2,000 years ago (median, 2,296 years; 95% confidence interval [CI], 1,428 to 3,340), which may reflect the loss of intermediate lineages over time or may represent two independent introductions of *B. pertussis* into the global human population from an unknown reservoir. The adaptation of *B. pertussis* to the human population has been postulated to have involved a significant evolutionary bottleneck and was associated with considerable gene loss and gene inactivation due to insertion sequence (IS) element expansion and mutations (30), a process commonly seen in host-restricted bacteria (31). In the analysis of the Tohama I genome sequence, it was estimated that up to 25% of genes were lost relative to those present in the common ancestor with *Bordetella parapertussis* (30), and 9.5% of those remaining were inactivated and were only present as pseudogenes. A manual comparison of 50% of the pseudogenes in Tohama I and strain 18323 (representing the two deep branches) showed that 72% of the pseudogenes were shared, and of those, all had identical inactivating mutations (Table S3). This indicates that the host restriction of *B. pertussis* and the associated bottleneck occurred before the divergence of these two lineages and long before the first description of the disease. The most parsimonious explanation would suggest that this process involved adaptation to the human host, and this would indicate that pertussis was introduced into the global population twice from a reservoir in an unsampled human population or that the intermediate diversity has been lost. The alternative explanation, that the adaption was to another host, would require both an unknown reservoir species and two separate introductions into the human population.

**TABLE 1:** Geographic origin and period of isolation of *B. pertussis* strains used in this study

Continent	Country	No. of strains	Isolation period	Introduction of vaccination
Africa	Kenya	17	1975	1980s
	Senegal	4	1990-1993	1980s
Asia	China	2	1957	Early 1960s
	Hong Kong	5	2002-2006	1950s
	Japan	17	1988-2007	1950s
	Taiwan	23	1992-2007	1954
Australia	Australia	37	1974-2007	1953
Europe	Denmark	9	1962-2007	1961
	Finland	16	1953-2006	1952
	France	11	1993-2007	1959
	Italy	15	1994-1995	1995
	Netherlands	60	1949-2010	1953
	Poland	16	1963-2000	1960
	Russia	2	2001-2002	1956-1959
	Sweden	23	1956-2006	1953
	United Kingdom	20	1920-2008	1957
North America	Canada	17	1994-2005	1943
	USA	36	1935-2005	1940s
South America	Argentina	13	1969-2008	1970s
Total		343	1920-2010	

Three vaccine strains were included in this study, Tohama I and two American strains (strains B308 and B310) (see Table S1 in the supplemental material), which were placed in branch II. The vaccine strains and the reference genome, Tohama I, both represent lineages and antigenic genotypes for which recent isolations are rare. Most recent *B. pertussis* isolates stem from a lineage (lineage IIb) within branch II, which appeared before the introduction of vaccination but has expanded since. A Bayesian phylogenetic and skyline analysis of isolates from lineage IIb for which isolation date information was available (Fig. 1B; Fig. S2) reveals that there was no evidence of loss of diversity (represented by the effective population size) after the introduction of vaccination. This was unexpected, as one would assume that the introduction of vaccination would lead to a decrease in population diversity, as the selective pressure may lead to a population bottleneck whereby only those lineages that escape the vaccine may survive. Indeed, some previous studies have observed such a decrease in population diversity following the introduction of vaccination. However, these studies were based on geographically more restricted pathogen populations (32–34). Our results suggest that, despite whole-cell vaccines reducing the prevalence of many of the older lineages, they have not been eradicated completely, so the diversity of these lineages is still present in the *B. pertussis* population. The explanation for this may be that such lineages have persisted in geographical regions where vaccination has not become routine. In fact, there is some evidence from the skyline analysis that population diversity increased in lineage IIb after vaccine introduction. Although the effect of sampling density before and after vaccine introduction is unclear, the shape of the phylogenetic tree suggests that this increase was primarily the result of the expansion of the *ptxA1* lineage, which may represent some level of vaccine escape in countries where vaccination had been introduced.

A second increase in effective population size (diversity) coincides with the emergence and expansion of a lineage carrying the *ptxP3* allele. In the mid-1990s, there appears to be a drop in diversity, correlating with the loss of a number of early *ptxA1* lineages, and perhaps corresponding with the introduction of the ACV in the mid-1990s. However, diversity very quickly increased again with the expansion of a sublineage of the *ptxP3* group that acquired a *fim3-2* allele, again suggesting the selection and diversification of vaccine escape lineages.

There is little evidence of geographical structure in the phylogenetic tree (Fig. 1B). The sampling of the older branch I lineages is sparse in space and time, making inference difficult. However, the *ptxA1* lineage is clearly dispersed globally, and the *ptxP3* and *fim3-2* lineages show no geographical clustering at all, indicating that there has been very rapid global spread of these recently evolved lineages.

### Temporal trends in frequencies of alleles coding for vaccine components.

To explore the influence of vaccination on the *B. pertussis* population, we focused on genes coding for antigens known to induce protection and included in modern ACVs, including serotype 2 fimbriae (*fim2*), serotype 3 fimbriae (*fim3*), pertactin (*prn*), and the A subunit of Ptx (*ptxA*) (5, 35). Although it is used in ACVs, filamentous hemagglutinin was not included, as accurate assembly and assignment of SNPs was not possible due to the presence of repeats and paralogs. As previous studies suggest that variation in the Ptx promoter, *ptxP*, was linked to clonal sweeps (15, 21, 33), we also included *ptxP* alleles in our analyses. With the exception of *ptxA10*, *prn16*, and *prn17*, all alleles have been described before, and references and accession numbers are given in Text S1 in the supplemental material. The major changes in antigen gene alleles (from *ptxA2* to *ptxA1*, *fim2-1* to *fim2-2*, *ptxP1* to *ptxP3*, and *fim3-1* to *fim3-2*) are marked on the nodes in the phylogenetic tree in Fig. 1B. In most countries, vaccination was introduced between 1940 and 1960 (Table 1), and worldwide, many different *B. pertussis* strains have been used to produce vaccines. A compilation of 23 vaccine strains revealed that the most prevalent alleles found in vaccine strains were *fim2-1* (82%), *fim3-1* (100%), and *prn1* (74%) or *prn7* (22%) (Table S4). If one Dutch, one Swedish, and one acellular vaccine strain were omitted, all other vaccine strains carried the *fim2-1*, *fim3-1*, and *prn1*/*7* alleles. More diversity in vaccine strains was observed with respect to *ptxA*, for which four alleles, *ptxA1*, *ptxA2*, *ptxA3*, and *ptxA4*, were observed at frequencies of 13%, 52%, 4%, and 31%, respectively. For twelve vaccine strains, the *ptxP* allele has been determined. The *ptxP1* allele and *ptxP2* allele were found in 67% and 33%, respectively. Most ACVs are derived from two strains, Tohama I and 10536, which carry the alleles *fim2-1*, *fim3-1*, *prn1*, *ptxA2*, and *ptxP1* and *fim2-1*, *fim3-1*, *prn7*, *ptxA4*, and *ptxP2*, respectively.

**FIG 2:**
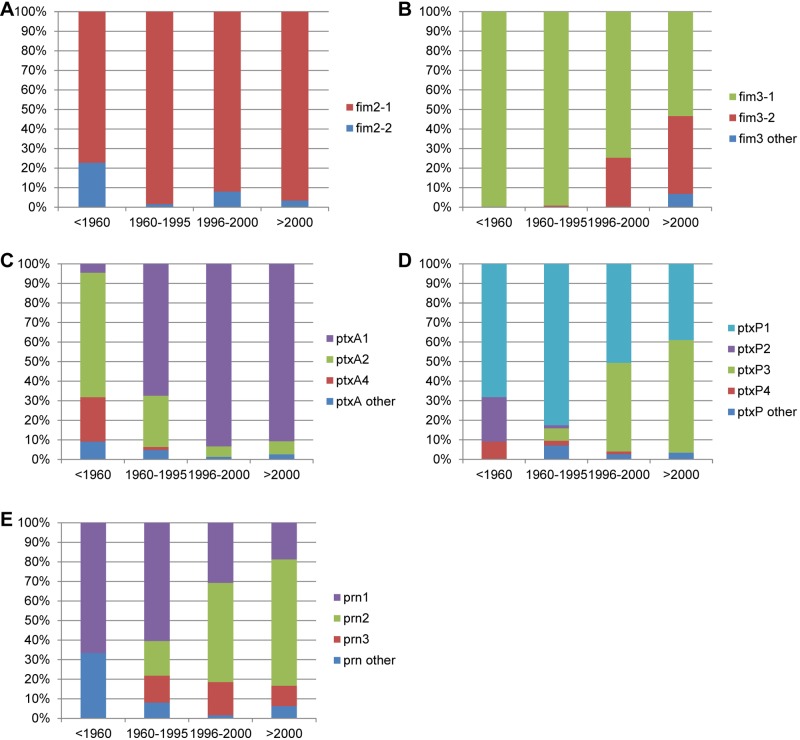
Temporal trends in strain frequencies for the *fim2* (A), *fim3* (B), *ptxA* (C), *ptxP* (D), and *prn* (E) alleles. Four periods were defined to reflect the worldwide changes in pertussis vaccination, the early WCV period (earlier than 1960), the period in which mainly WCVs were used (WCV period, 1960 to 1995), the period in which both WCVs and ACVs were used (WCV/ACV period, 1996 to 2000), and a period in which mainly ACVs were used (ACV period, later than 2000).

To investigate temporal trends in allele frequencies, we defined four periods to reflect the worldwide changes in pertussis vaccination (Fig. 2): the early WCV period (earlier than 1960; *n* = 22), the period in which mainly WCVs were used (WCV period, 1960 to 1995; *n* = 126), the period in which both WCVs and ACVs were used (WCV/ACV period, 1996 to 2000; *n* = 75), and finally, a period in which mainly ACVs were used (ACV period, later than 2000; *n* = 118). We presumed that the effect of vaccination on the *B. pertussis* population was small in the early WCV period (15, 33). Obviously, the relationship between the periods and the vaccination history can only be approximate.

Two *fim2* alleles were observed in the worldwide collection of strains, *fim2-1* (the vaccine type) and *fim2-2*, the products of which differed in a single amino acid. The *fim2-1* allele predominated in all four periods (frequencies 77% to 98%), whereas the *fim2-2* allele was found at low frequencies (2% to 23%) in all four periods (Fig. 2A). Phylogenetic analysis (Fig. 1B) indicated that the mutation leading to the *fim2-2* allele arose twice within lineage IIb but also occurred on the branch leading to lineage IIa. Bayesian analysis suggested that, within lineage IIb, the mutation occurred between 1970 and 1984 (95% CI, 1956 to 1992) on the first occasion and between 1996 and 2002 (95% CI, 1995 to 2002) on the second. Thus, the first mutation arose in the WCV period and the most recent mutation occurred in the WCV/ACV period.

More variation was found in *fim3*, for which five alleles were identified. As one allele contains a silent mutation, the five alleles code for four distinct proteins: Fim3-1, Fim3-2, Fim3-3, and Fim3-6. The *fim3-1* (the vaccine type) and *fim3-2* alleles were predominant (Fig. 2B). The polymorphic amino acid residue in *fim3-2* relative to the sequence of *fim3-1* is located in a surface epitope that has been shown to interact with human serum (36). The *fim3-1* allele has always predominated, but the *fim3-2* allele, which was first detected in the WCV period (frequency 1%), increased in frequency to 37% in the ACV period. Our analyses agreed with this observation, with the mutation resulting in the *fim3-2* allele predicted to have occurred between 1986 and 1989 (95% CI, 1982 to 1992).

Eight *ptxA* alleles were found worldwide, two of which contained silent mutations. Thus, the eight alleles resulted in six protein variants (PtxA1, PtxA3, PtxA4, PtxA5, PtxA9, and PtxA10), mostly differing by one or two amino acids. Three alleles were predominant, *ptxA1*, *ptxA2*, and *ptxA4* (respective frequencies, 78%, 18%, and 2%). The *ptxA2* and *ptxA4* alleles predominated in the early WCV period (respective frequencies, 64% and 23%). Our analyses show that the *ptxA1* allele arose between 1921 and 1932 (95% CI, 1905 to 1942), before the introduction of vaccination. It increased in frequency from only 5% in the early WCV period to 68%, 92%, and 90% in subsequent periods (Fig. 2C). Although most (46%) of the vaccine strains harbor *ptxA2*, 17% do contain *ptxA1*.

Fourteen *ptxP* alleles were observed, of which *ptxP1* and *ptxP3* predominated (total frequencies of 60% and 32%, respectively). Strains with *ptxP1* were most common in the early WCV and WCV periods (respective frequencies, 68% and 83%) but were replaced by *ptxP3* strains in the last two periods (the *ptxP3* frequencies in the WCV/ACV and ACV periods were 48% and 57%, respectively) (Fig. 2D). Bayesian analysis suggested that the mutation resulting in the *ptxP3* allele arose between 1974 and 1977 (95% CI, 1970 to 1981), i.e., in the WCV period.

Twelve *prn* alleles were identified, of which 11 led to protein variants (Prn1 to -7, Prn10 to -12, and Prn16). Prn-deficient strains were not detected, presumably because these strains reached significant frequencies in a later period than analyzed in this study. Three alleles predominated in our worldwide collection, *prn1* (42%), *prn2* (38%), and *prn3* (12%). In the early WCV period, 67% of the strains harbored *prn1* (the vaccine type), with *prn2* and *prn3* alleles emerging in the WCV period. While the frequency of the *prn3* allele remained more or less constant (10% to 17%), *prn2* increased in frequency from 18% in the WCV period to 65% in the ACV period (Fig. 2E). Variation in *prn* mainly occurs by variation in numbers of repeats, a reversible process which is relatively frequent compared to point mutations. Therefore, many *prn* variants were homoplasic in our tree due to convergent evolution.

In conclusion, based on these five genes, it appears that the worldwide *B. pertussis* population has changed significantly in the last 60 years, consistent with other studies using temporally and geographically less diverse collections (15, 17–19, 21, 22, 32, 34, 37–40). Most changes resulted in genetic divergence from vaccine strains, consistent with vaccine-driven immune selection. Indeed, Bayesian analyses suggested that the non-vaccine-type alleles *ptxP3* and *fim3-2* arose in the period in which the WCV was used widely. Recently, strains have been identified which do not express Prn and/or FHA (17, 23, 24), and the emergence of these strains may be associated with the introduction of ACVs. In this and previous work, the largest number of alleles were observed for *ptxP* (*n* = 14), *prn* (*n* = 12), and *ptxA* (*n* = 8). The number of alleles may be related to the degree of diversifying selection caused, e.g., by the immune status of the host population or other (frequent) changes in the ecology of *B. pertussis*.

Previous studies have shown that changes in *fim3*, *ptxA*, *prn*, and *ptxP* are associated with selective sweeps (15, 19, 22, 32), implying a significant effect on strain fitness. Furthermore, variation in *ptxA*, *ptxP*, and *prn* has been shown to affect bacterial colonization of naive and vaccinated mice (40–45), underlining the biological significance of these changes. However, in one study, the effects were not observed (46).

### Identification of additional loci potentially involved in adaptation.

In addition to focusing on genes coding for vaccine components, we used a more comprehensive approach to identify putative adaptive loci. To detect genes important for adaptation, *dN*/*dS* ratios (ratio of nonsynonymous to synonymous substitution rates) are widely used. This method was originally developed for the analysis of divergent species and needs a large number of substitutions for a statistically reliable analysis (47–49). However, *B. pertussis* strains are highly related and differ by less than 0.1% in their genomic sequences. Recent studies have shown that the primary driver of *dN*/*dS* ratios in such closely related strains is time, not selection (48). Furthermore, the approach using *dN*/*dS* ratios assumes that silent mutations are neutral. However, silent mutations in genes can significantly affect gene expression (50). Finally, *dN*/*dS* ratios are not useful to detect diversifying selection in intergenic regions. Therefore, we chose to assess diversifying selection by focusing on SNP densities and homoplasy.

**FIG 3:**
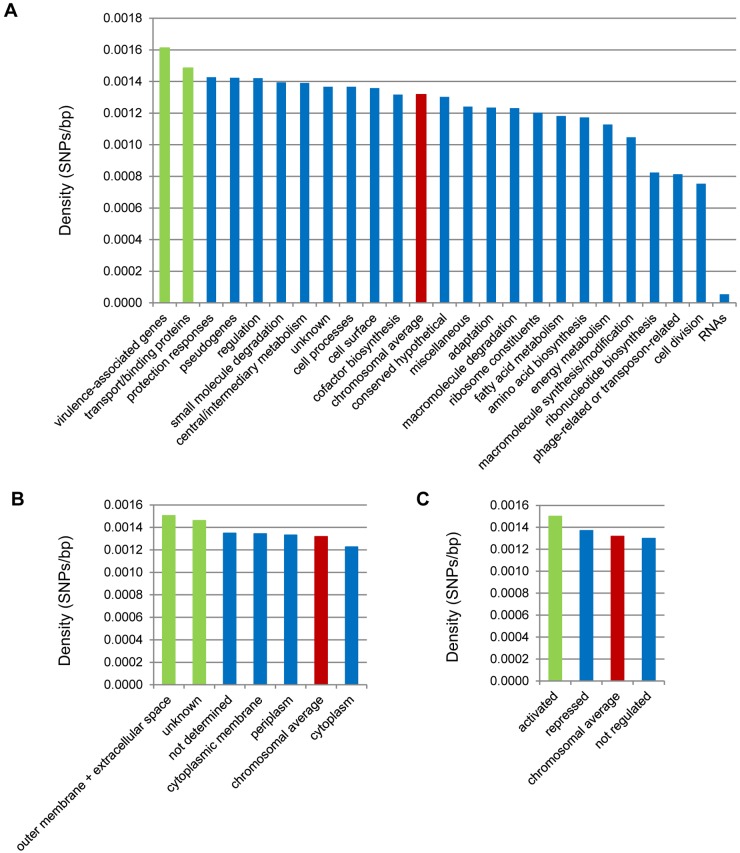
SNP densities per functional category (A), subcellular localization (B), and Bvg regulation (C). Red bars indicate the chromosomal average. Green bars refer to categories with an SNP density significantly higher than the chromosomal average (*P* < 0.05).

**SNP densities.** We explored whether particular gene categories had a significantly higher SNP density than the overall SNP density of the whole genome, 0.0013 SNPs/bp. The gene categories used were defined by Parkhill et al. (30), with modifications, i.e., pseudogenes and genes known or assumed to be associated with virulence were placed in separate categories. In all, 24 gene categories were defined (Fig. 3A; see Table S5 in the supplemental material). As expected, gene categories involved in housekeeping functions, which are generally conserved, showed the lowest SNP densities (0.0007 to 0.00012 SNPs/bp). The four categories with the highest SNP density were virulence associated (0.0016 SNPs/bp), transport/binding (0.0015 SNPs/bp), protection responses (0.0014 SNPs/bp), and pseudogenes (0.0014 SNPs/bp), which are likely to be evolving neutrally since their inactivation. Only for the virulence-associated and transport/binding categories did the SNP density difference reach statistical significance, however (*P* = 0.02 and *P* = 0.03, respectively). The high SNP density in the transport/binding category was surprising, as this category mostly codes for housekeeping functions, including transport of molecules such as amino acids, small ions, and carbohydrates. The high SNP density may reflect changes in the physiology of *B. pertussis* or the surface exposure of membrane and periplasmic components of these systems.

To investigate this further, we tested whether the subcellular location of proteins would result in significantly different degrees of SNP density, as surface-exposed proteins are expected to be subject to a higher degree of immune selection than intracellular proteins. In line with this, we found that if categories were based on subcellular location prediction, genes coding for proteins exposed to the host environment (extracellular and outer membrane proteins) had the highest SNP density (0.0015 SNPs/bp; *P* = 0.05), whereas genes coding for cytoplasmic proteins showed the lowest SNP density (0.0012 SNPs/bp; *P* = 1.0) (Fig. 3B; see Table S5 in the supplemental material). In addition to the exposed category, only the category “unknown,” which comprises proteins for which we could not predict a location, showed an SNP density which was significantly higher than the genomic average (0.0015 SNPs/bp; *P* = 0.007). For example, Ptx subunits 2 to 5 are included in the unknown category, although it is known that they are secreted (51). Possibly this category compromises more genes that encode surface-exposed proteins but for which the location could not be predicted.

We also assessed the SNP density in gene categories based on Bvg regulation (26, 27). For this, three categories were defined: genes activated, repressed, or unaffected by Bvg (Fig. 3C; see Table S5 in the supplemental material). The SNP density in these three categories decreased in the order Bvg activated, Bvg repressed, and not regulated by Bvg (SNP densities, 0.0015, 0.0014, and 0.0013 SNPs/bp, respectively; *P* = 0.013, *P* = 0.40, and *P* = 1.0, respectively). The relatively high SNP density in Bvg-activated genes was not unexpected, as genes encoding virulence-associated proteins and extracellular proteins are included in this category.

Focusing on gene categories increased the power of the statistical analyses but only gave a general picture and did not reveal individual loci that might be under selection. Therefore, we also identified particular loci which were highly polymorphic. For this, we calculated whether there was an overrepresentation of SNPs in a locus given its length (Table 2; see Table S5 in the supplemental material). Two genes showed a significantly higher SNP density than the chromosomal average of 0.0013 SNPs/bp in genes. One gene encodes Ptx subunit A (*ptxA*) (0.011 SNPs/bp; *P* = 0.0033). The other gene, *cysB* (0.011 SNPs/bp; *P* = 0.0029), encodes a LysR-like transcriptional regulator that acts as an activator of the *cys* genes and plays a role in sulfur metabolism (52, 53).

**TABLE 2:** Genes and promoters with SNP densities significantly higher than the chromosomal average

Locus tag(s)	Gene(s)	Density (SNPs/bp)	*P* value	Product	Category^*a*^	Localization(s)^*b*^	Bvg^*c*^
3783BP	*ptxA*	0.01111	3.3E-03	Pertussis toxin subunit A precursor	Vir	E	+
2416BP	*cysB*	0.01053	2.9E-03	LysR family transcriptional regulator	Reg	C	
BP3783P	*ptxP*	0.07143	4.7E-18	Pertussis toxin promoter	Vir	E	+
BP2936P		0.03623	2.0E-02	Putative methylase promoter	Exp	CM	+
BP1878P, BP1879P	*bvgP*, *fhaBP*	0.02582	3.4E-05	Virulence factor transcription regulator promoter, filamentous hemagglutinin	Vir	C, OM	+, +
BP3723P, BP3724P		0.02047	1.8E-02	Hypothetical protein promoter	Hyp	U, C	

^a^ Functional category: Vir, virulence-associated genes; Reg, regulation; Exp, exported proteins; Hyp, hypothetical proteins.

^b^ Subcellular localization: E, extracellular; C, cytoplasmic; CM, cytoplasmic membrane; OM, outer membrane; U, unknown.

^c^ Regulation by Bvg: +, activated; blank cells, not activated or repressed.

We also investigated SNP densities in intergenic regions, as these may be involved in transcription of downstream genes. We found four putative promoter regions with a significantly higher SNP density than the chromosomal average of 0.0026 SNPs/bp in intergenic regions (Table 2; see Table S5 in the supplemental material). Two promoter regions were located upstream from virulence-associated genes. One was upstream from the *ptx* operon (0.071 SNPs/bp; *P* = 4.7 × 10^−18^), and one was between the filamentous hemagglutinin gene (*fhaB*) and the *bvg* operon (0.026 SNPs/bp; *P* = 3.4 × 10^−5^). The extensive polymorphism in the Ptx promoter has been described previously (14, 16). Eleven SNPs were located in the intergenic region between the *bvg* operon and *fhaB*, which has been studied extensively (54–58). Seven and four SNPs were located in regions assumed to affect the transcription of *fhaB* and *bvgA*, respectively (Text S2). While the SNPs in the *fhaB* promoter may affect the expression of both *fha* and *fim* genes, which are part of a single operon (59), the SNPs in the *bvgA* promoter region may have a significant effect on the expression of many virulence factors. A high SNP density was also observed in the region upstream from a putative methylase possibly involved in ubiquinone/menaquinone biosynthesis (0.036 SNPs/bp; *P* = 0.020) and in the promoter region of two hypothetical proteins (0.020 SNPs/bp; *P* = 0.018).

In conclusion, we identified significantly higher SNP densities in virulence-associated genes, genes encoding surface-exposed proteins, and genes activated by Bvg. High SNP densities were also observed in the promoter regions for *ptx* and *bvg*/*fha*. The finding of a high SNP density in *cysB* was interesting, as a number of associations have been observed between sulfur metabolism and virulence (60). Indeed, in *B. pertussis*, the expression of virulence-associated genes is affected by the sulfate concentration (28). The identification of putative adaptive loci allows focused studies that may reveal novel strategies for pathogen adaptation.

### Homoplasic SNPs.

In a second approach to find loci possibly involved in adaptation, we identified homoplasic SNPs, that is, SNPs which arose independently on different branches of the tree. In our data set, 15 SNPs were homoplasic (Table 3). Thirty-three percent of the homoplasic SNPs were located in Bvg-activated genes, while this category only comprises 6% of the genome. The 5 SNPs found in Bvg-activated genes were located in genes for the serotype 2 and 3 fimbrial subunits (*fim2* and *fim3*), a type III secretion protein (*bscI*), a Ptx transport protein (*ptlB*), and a periplasmic solute-binding protein (*smoM*) involved in transport of mannitol. Of the remaining 10 homoplasic SNPs, 6 and 4 were located in genes and intergenic regions, respectively. Remarkably, one homoplasic SNP found in *cysM* was observed in five branches. The *cysM* gene codes for cysteine synthase, which is involved in cysteine biosynthesis and sulfate assimilation. All other homoplasic SNPs occurred in two branches.

**TABLE 3:** Homoplasic SNPs

Position^*a*^	Locus tag(s)	Gene	Branches^*b*^	Bootstrap^*c*^	Change^*d*^	Product (distance to ATG in bp)	Functional category	Localization^*e*^	Bvg^*f*^
612075	BP0607	*gpm*	2 (1, 3)	99	Silent	Phosphoglycerate mutase 1	Energy metabolism	Cytoplasmic	
667028	BP0658		2 (1, 19)	55	Q30	Putative dehydrogenase	Miscellaneous	Cytoplasmic	
925864	BP0888		2 (7, 1)	100	Silent	GntR family transcriptional regulator	Regulation	Cytoplasmic	
997017	BP0958	*cysM*	5 (1, 1, 4, 2, 1)	100	G247E	Cysteine synthase B	Amino acid biosynthesis	Cytoplasmic	
1109310	1064BP	*maeB*	2 (6, 1)	100	Silent	NADP-dependent malic enzyme	Central/intermediary metabolism	Cytoplasmic	
1109312	1064BP	*maeB*	2 (6, 1)	100	Q28P	NADP-dependent malic enzyme	Central/intermediary metabolism	Cytoplasmic	
1175956	1119BP	*fim2*	2 (7, 9)	100	R177K	Serotype 2 fimbrial subunit precursor	Virulence- associated genes	Extracellular	+
1565529	1487BP	*smoM*	2 (1, 4)	100	R176K	Putative periplasmic solute-binding protein	Transport/binding proteins	Unknown	+
1647989	1568BP	*fim3*	2 (1, 1)	98	T130A	Serotype 3 fimbrial subunit precursor	Virulence- associated genes	Extracellular	+
2018882	BP1914P		2 (1, 2)	100	Intergenic	Transposase for IS*1663* (321)	Phage or transposon related	Unknown	
	BP1915P		2 (1, 2)	100	Intergenic	Conserved hypothetical protein (23)	Conserved hypothetical	Unknown	
2213448	BP2090P		2 (8, 1)	100	Intergenic	ABC transporter substrate-binding protein (306)	Transport/binding proteins	Periplasmic	−
	BP2091P		2 (8, 1)	100	Intergenic	Dioxygenase hydroxylase component (53)	Small molecule degradation	Cytoplasmic	−
2374322	2249BP	*bscI*	2 (1, 97)	60	Y114C	Type III secretion protein	Virulence- associated genes	Unknown	+
3041105	BP2862P		2 (6, 1)	100	Intergenic	Conserved hypothetical protein (174)	Unknown	Unknown	
	BP2863P		2 (6, 1)	100	Intergenic	Conserved hypothetical protein (148)	Unknown	Cytoplasmic	
3251279	BP3052P		2 (6, 2)	100	Intergenic	Putative gamma- glutamyl transpeptidase (242)	Miscellaneous	Periplasmic	
3992064	3789BP	*ptlB*	2 (1, 1)	69	Silent	Pertussis toxin transport protein	Virulence- associated genes	CM	+

^a^ Position in reference genome *B. pertussis* Tohama I.

^b^ Number of branches in which the homoplasic SNP occurred (number of strains/branch).

^c^ Number of trees in which SNP is homoplasic (100 trees tested).

^d^ Change in amino acid.

^e^ Subcellular localization: CM, cytoplasmic membrane.

^f^ Regulation by Bvg: + activated; − repressed; blank cells, not activated or repressed.

Convergent evolution is extremely rare in monomorphic bacteria like *B. pertussis*. In other monomorphic bacteria, homoplasy is usually only found in a few genes involved in antibiotic resistance (61). This suggests that the homoplasic SNPs we have identified may play an important role in the adaptation of *B. pertussis.*

### Gene loss.

Several studies have shown that some *B. pertussis* isolates contain DNA that is not in Tohama but is present in *Bordetella bronchiseptica* and *Bordetella parapertussis* (62–66). In this work, we performed a *de novo* assembly of all of the genomes and compared each assembly back against the reference Tohama I in order to identify any genomic DNA that may have been acquired since the origin of *B. pertussis*. This analysis showed no evidence of gene gain at any point in the phylogeny. All regions identified in the sample data set that were not in Tohama are present in other *Bordetella pertussis* genomes, such as 18323, consistent with gene loss in Tohama. Placing these regions onto the tree showed that progressive gene loss within multiple lineages can be observed (see Fig. S3 in the supplemental material).

### Summary.

With the determination of the global population structure of *B. pertussis* using whole-genome sequencing, we addressed key questions concerning the origin of pertussis, such as the forces that have driven the shifts in *B. pertussis* populations and the role of these shifts in the resurgence of pertussis. Despite a structure suggesting two relatively recent introductions of *B. pertussis* from an unknown reservoir, phylogenetic analysis did not reveal the ancient geographic origin of *B. pertussis*, possibly because rapid worldwide spread and selective sweeps have eliminated geographic signatures. Indeed, our results showed that the mutation that resulted in the *ptxP3* allele, which is associated with an increase in pertussis notifications in at least two countries (14, 20), occurred once and strains carrying this new allele spread worldwide in 25 to 30 years.

We confirmed and extended the observation that the worldwide *B. pertussis* population has changed significantly in the last 60 years, consistent with other studies using temporally and geographically less diverse collections (15, 17–19, 21, 32, 34, 37–40). We used several approaches to identify gene categories under selection, including SNP density and homoplasy. These approaches consistently suggested that Bvg-activated genes and genes coding for surface-exposed proteins were important for adaptation. At the individual gene level, four of the five genes for the components of current ACVs were found to be particularly variable, underlining their role in inducing protective immunity and consistent with vaccine-driven immune selection.

We identified other, less obvious genes which contained potentially adaptive mutations, such as two genes involved in cysteine and sulfate metabolism (*cysB* and *cysM*). Sulfate can be used to regulate virulence-associated genes *in vitro* (67), and our results suggest that sulfate may also be an important cue during natural infection. This result suggests that host-pathogen signaling and/or the physiology of *B. pertussis* has changed over time.

Temporal analyses showed that most mutations in genes encoding acellular vaccine components arose in the period in which the WCV was used. It should be noted, however, that the period in which the WCV was used (30 to 40 years) is much longer than the ACV period (7 to 15 years). These results are consistent with a significant effect of vaccination on the *B. pertussis* population, as suggested by previous studies (5, 20, 32, 39, 68). It seems plausible that the changes in the *B. pertussis* populations have reduced vaccine efficacy.

Pathogen adaptations may reveal weak spots in the bacterial defense, and hence, the loci under selective pressure may point to ways to improve pertussis vaccines. Furthermore, many of the putative adaptive loci we identified have a physiological role, and future studies of these loci may reveal less obvious ways in which the pathogen and host interact.

## MATERIALS AND METHODS

### Strains and sequencing.

The clinical isolates used in this study are listed in Table S1 in the supplemental material. DNA was isolated by the participants and sequenced using Illumina technology (69). Nineteen isolates were sequenced using the Genome Analyzer II and resulting in single reads of 37 bp (sequencing method 1). Thirty-eight isolates were sequenced using the Genome Analyzer II and resulting in paired-end reads of 50 bp (sequencing method 2). The remaining isolates were sequenced using 12 multiplexed tags on the Genome Analyzer II, producing paired-end reads of 54 bp (sequencing method 3). The accession numbers of the raw sequence data are listed in Table S1.

### SNP detection.

Reads for all sequenced samples were mapped against the complete Tohama I reference genome sequence (accession number BX470248) using SMALT (http://www.sanger.ac.uk/resources/software/smalt/). Reads mapping with identical matches to two regions of the reference genome were left unmapped. The alignment of reads around insertions and deletions (indels) was improved using a combination of pindel (70) to identify short indels and dindel (71) to realign the reads. SNPs were identified using samtools mpileup (http://samtools.sourceforge.net) and filtered as described previously (72)

Information about promoters, genes, and proteins was retrieved from the sequenced genome of *B. pertussis* Tohama I. The annotation was updated using BLAST (73), and domain information was recovered from SMART (74) and Conserved Domain Database (75).

Homoplasic SNPs were identified by reconstructing base changes for each variable site onto the phylogenetic tree under the parsimony criterion. Any site for which the observed number of base changes for the maximum parsimony reconstruction on the tree was greater than the minimum possible number of changes for that site is homoplasic.

### Phylogeny.

The phylogenetic relationships of the entire data set were inferred under a maximum likelihood framework using PHYML (76) with an HKY85 model of evolution. The global phylogeny was rooted using *B. bronchiseptica* MO149 (sequence type 15 [ST15]), which was previously shown to be most closely related to *B. pertussis* (77, 78).

Mutation rates and ancestral node dates for lineage IIb were estimated using Bayesian analysis in the BEAST version 1.6.2 package (79). Analyses using the variable sites within lineage IIb isolates with isolation dates available were run under a general time reversible (GTR) model of evolution, with all combinations of constant, expansion, logistic and skyline population size models, and strict, relaxed exponential, and relaxed log-normal clock models. For each combination, three independent Markov chains were run for 100 million generations each, with parameter values sampled every 1,000 generations. Chains were manually checked for reasonable ESS values and for convergence between the three replicate chains using Tracer. Tracer was also used to identify a suitable burn-in period to remove from the beginning of each chain, as well as to assess the model with the best fit to the data using Bayes factors. A skyline population model with a relaxed exponential clock model was identified as the most appropriate, so this combination of models was used for all further analyses. It was found that, in each case, a burn-in of 10 million generations was clearly past the point where chains appeared to have converged, so this was chosen as the burn-in for all chains. The burn-in was removed and chains combined and down-sampled to every 10,000 generations using LogCombiner. A Bayesian skyline plot was calculated in Tracer using the default parameters, and a maximum clade credibility tree computed with TreeAnnotator.

### SNP densities.

The functional categories used were defined by Parkhill et al. (30), with modifications, i.e., pseudogenes and genes known or assumed to be associated with virulence were placed in separate categories. Subcellular localization was predicted by PSORTb version 3.0 (80). Bvg categories were defined based on the results of Streefland et al. (27) and Cummings et al. (26). For the length of a specific category or locus repeat, regions were excluded because SNPs in these regions are not reliable. To determine the number of bases in a specific category, the lengths of the included loci were added, excluding repeat regions. To determine whether the SNP density of a particular group or locus was significantly higher than the chromosomal average, Fisher’s exact test was used. *P* values were corrected according to the method of Benjamini and Hochberg (81).

## SUPPLEMENTAL MATERIAL

Table S1 Characteristics of 343 *B. pertussis* strains used in this study. Table S1, XLSX file, 0.1 MB.

Table S2 List of SNPs identified in this study. Table S2, XLSX file, 0.1 MB.

Table S3 Pseudogenes shared by Tohama and 18323. Table S3, XLSX file, 0.1 MB.

Table S4 Overview of strains used for production of pertussis vaccines. Table S4, XLSX file, 0.2 MB.

Table S5 SNP densities per functional category, subcellular location, Bvg regulation, gene, and intergenic region. Table S5, XLSX file, 0.5 MB.

Figure S1 Maximum likelihood phylogenetic tree of all isolates included in this study (see Table S1). (A) The complete tree of all isolates rooted on a ST15 *B. bronchiseptica* isolate, strain MO149. The branch leading to MO149 has been shortened to allow the relationships within *B. pertussis* to be seen. (B) A zoomed view of the maximum likelihood tree of lineage II to allow the relationships in this lineage to be more clearly seen. Scale bars indicate numbers of SNPs. Download Figure S1, PDF file, 0.4 MB

Figure S2 Bayesian skyline plot of *B. pertussis* illustrating variation in effective population size of lineage IIb over time. Download Figure S2, PDF file, 0.2 MB

Figure S3 Gene loss in *B. pertussis* isolates compared to Tohama I (A) and the distribution of accessory contigs not present in Tohama I (B). Download Figure S3, PDF file, 0.5 MB

Text S1 Overview of alleles coding for antigens known to induce protection and included in modern acellular pertussis vaccines. Download Text S1, DOCX file, 0.1 MB

Text S2 Polymorphisms in the *bvgA* and *fhaB* promoter region. Download Text S2, PDF file, 0.1 MB
