# Supplementary material for: Global Population Structure and Evolution of Bordetella pertussis and Their Relationship with Vaccination
Source: mBio. 2014 Apr 22;5(2):e01074-14. doi: 10.1128/mBio.01074-14 (PMC3994516; doi:10.1128/mBio.01074-14)

Supplemental File S5. Bayesian skyline plot of *B. pertussis* illustrating variation in effective population size of the major clade in lineage II over time. Above the plot is the associated Bayesian phylogeny from Figure 1. The column of coloured bars alongside the tree indicates contient of origin of the sample, as in Figure 1.

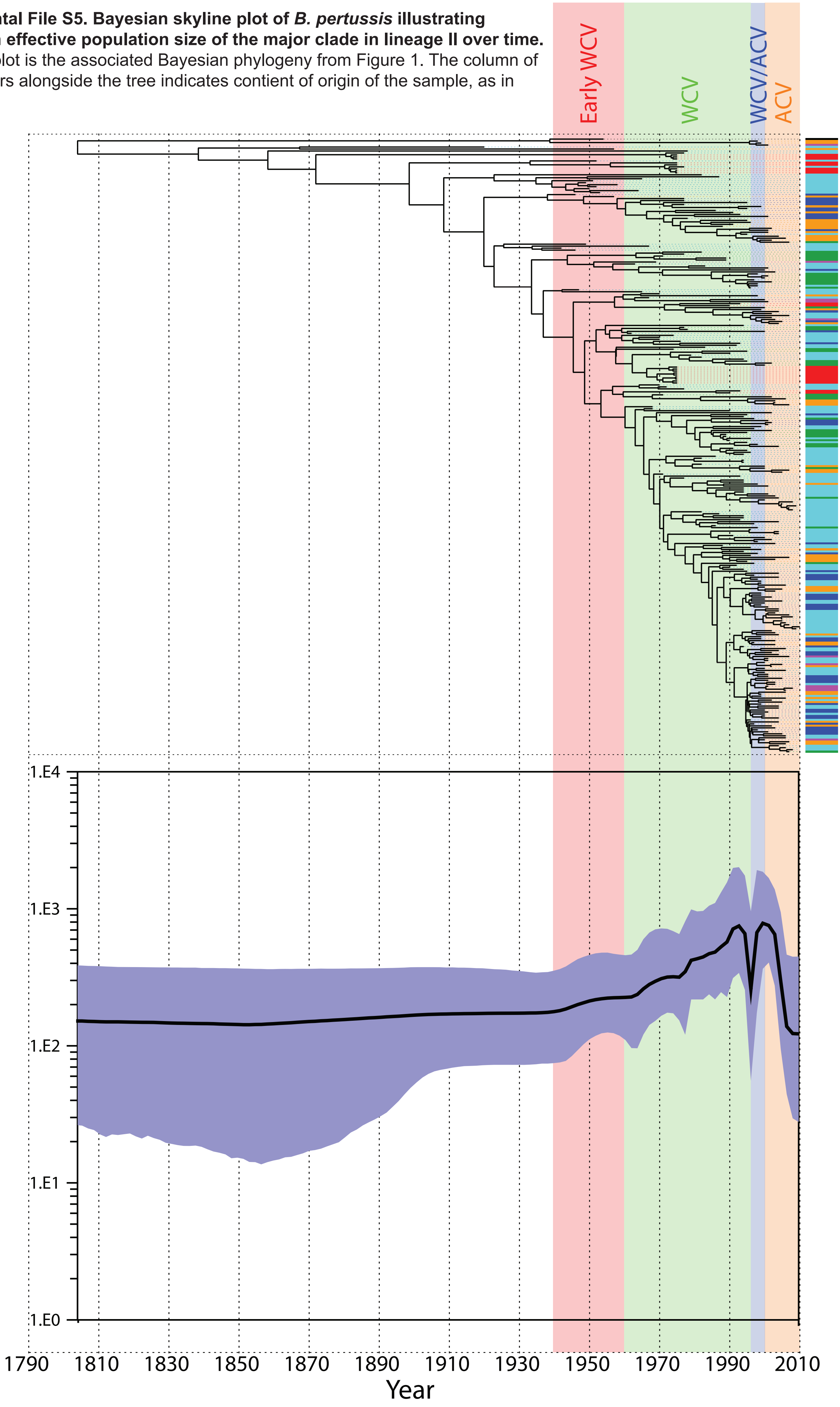

Supplement: Figure S2 — Bayesian skyline plot of B. pertussis illustrating variation in effective population size of lineage IIb over time. Download [file mbo002141804sd5.pdf]
