# Supplementary material for: Global Population Structure and Evolution of Bordetella pertussis and Their Relationship with Vaccination
Source: mBio. 2014 Apr 22;5(2):e01074-14. doi: 10.1128/mBio.01074-14 (PMC3994516; doi:10.1128/mBio.01074-14)

**Supplemental File S10. Gene loss in *B. pertussis* isolates compared to Tohama I (A) and the distribution of accessory contigs not in Tohama I (B)**

A

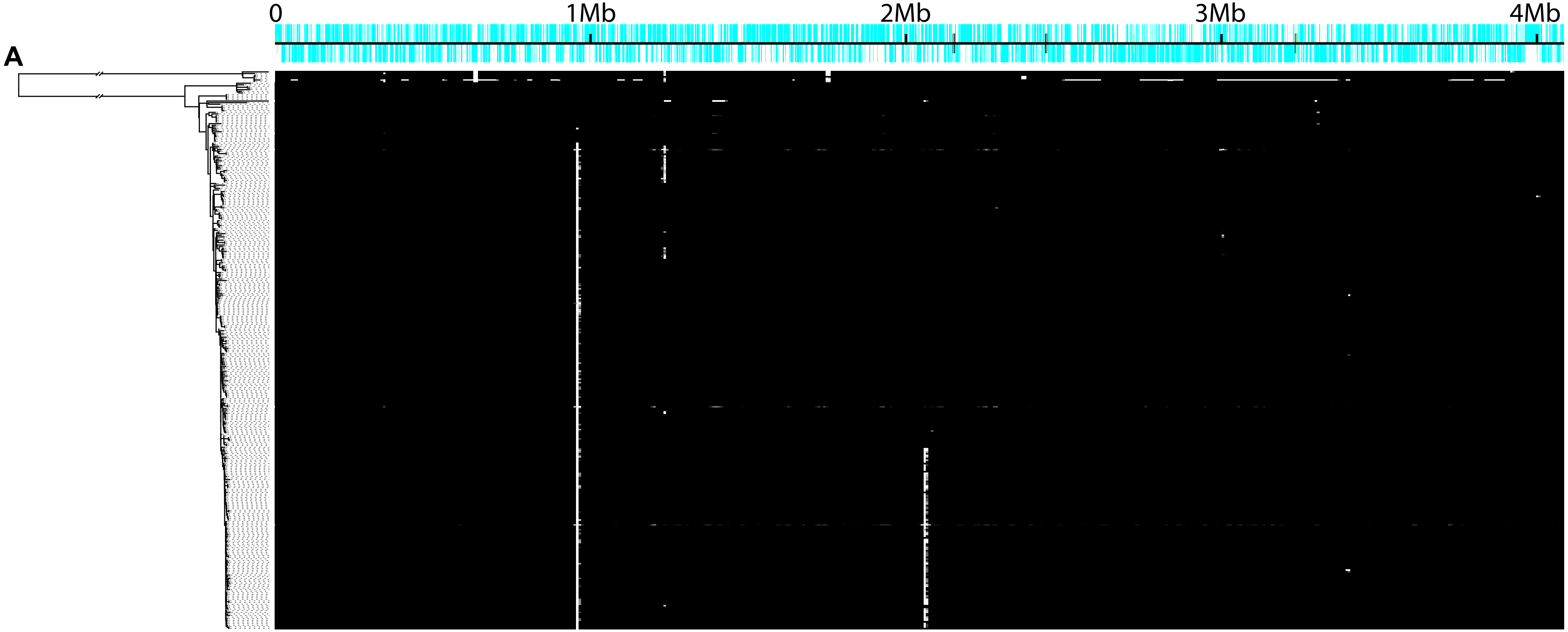


B

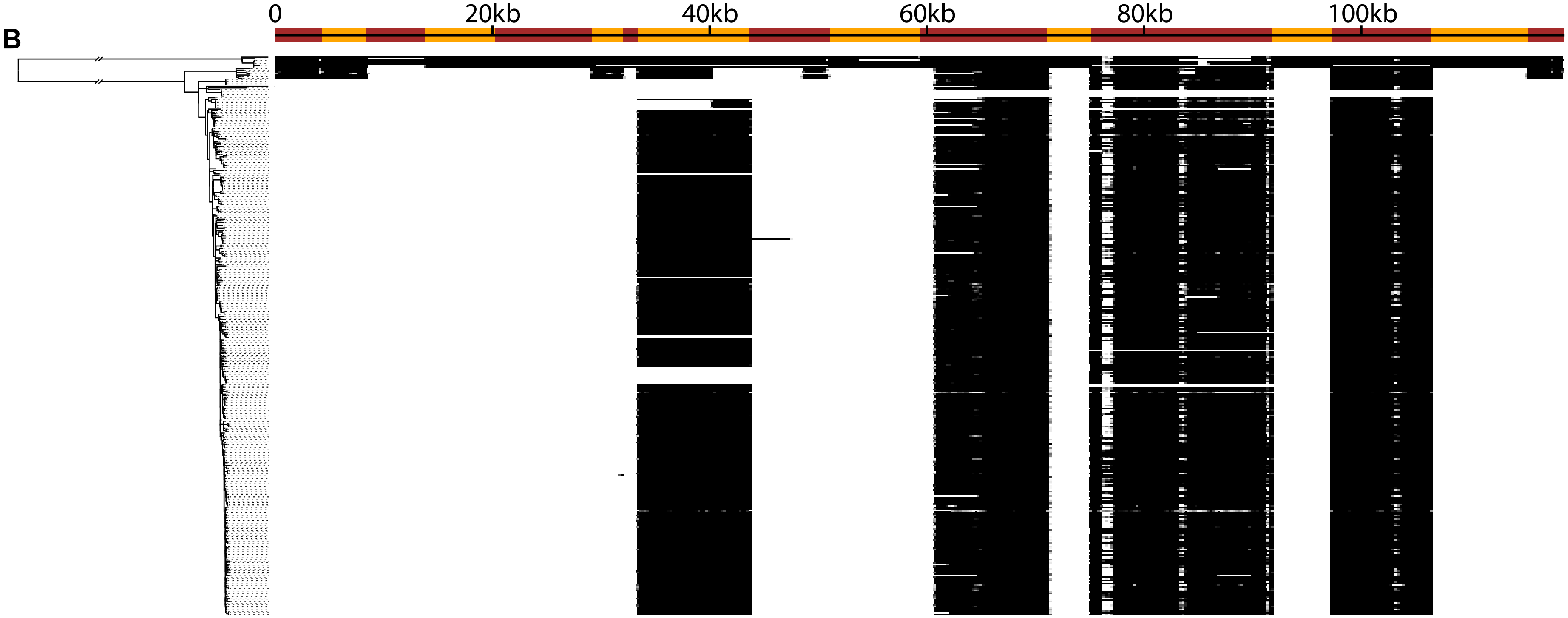

Supplement: Figure S3 — Gene loss in B. pertussis isolates compared to Tohama I (A) and the distribution of accessory contigs not present in Tohama I (B). Download [file mbo002141804sd10.pdf]
