# Supplementary material for: Global Population Structure and Evolution of Bordetella pertussis and Their Relationship with Vaccination
Source: mBio. 2014 Apr 22;5(2):e01074-14. doi: 10.1128/mBio.01074-14 (PMC3994516; doi:10.1128/mBio.01074-14)
Supplement: Text S1 — Overview of alleles coding for antigens known to induce protection and included in modern acellular pertussis vaccines. Download [file mbo002141804sd6.docx]

**Supplemental File S6. Overview of alleles coding for antigens known to induce protection and included in modern acellular pertussis vaccines**

| **Allele** | **Accession number** | **Reference** | **Remarks** |
| --- | --- | --- | --- |
| fim2-1 | Y00527 | (van Loo, Heuvelman et al. 2002) |  |
| fim2-2 | AJ420989 | (van Loo, Heuvelman et al. 2002) |  |
| fim3-1 | X51543 | (Tsang, Lau et al. 2004) | fim3A |
| fim3-2 | AY464180 | (Tsang, Lau et al. 2004) | fim3B |
| fim3-3 | AY464181 | (Tsang, Lau et al. 2004) | fim3C |
| fim3-4 | AY464179 | (Tsang, Lau et al. 2004) | fim3A* |
| fim3-5 | CAA52217 | (van Gent, Bart et al. 2012) |  |
| fim3-6 | JX100833 | (Shuel, Jamieson et al. 2013) | fim3E |
| prn1 | AJ011091 | (Mooi, van Oirschot et al. 1998) |  |
| prn2 | AJ011092 | (Mooi, van Oirschot et al. 1998) |  |
| prn3 | AJ011093 | (Mooi, van Oirschot et al. 1998) |  |
| prn4 | AJ011015 | (Mooi, He et al. 1999) |  |
| prn5 | AJ011016 | (Mastrantonio, Spigaglia et al. 1999) |  |
| prn6 | AJ132095 | (Mooi, van Oirschot et al. 1998) |  |
| prn7 | AJ133784 | (Mooi, van Oirschot et al. 1998) |  |
| prn8 | AJ133245 | (Mooi, van Oirschot et al. 1998) |  |
| prn9 | AJ315611 | Heuvelman, Peppler, Lewandowski, Mooi. Unpublished |  |
| prn10 | AJ784875 | (van Loo, Heuvelman et al. 2002) |  |
| prn11 | AJ507642 | (Poynten, McIntyre et al. 2004) |  |
| prn12 | AB278117 | (Han, Kamachi et al. 2008) |  |
| prn13 | EF486277 | Mooi, van der Heide, van Amersfoorth, Hallander. Unpublished |  |
| prn14 | HQ165753 | (Schmidtke, Boney et al. 2012) |  |
| prn15 | JX100834 | (Shuel, Jamieson et al. 2013) |  |
| prn16 | KC981248 | This work |  |
| prn17 | KC981249 | This work |  |
| ptxA1 | AJ245366 | (Mooi, van Oirschot et al. 1998) | ptxS1A |
| ptxA2 | AJ245367 | (Mooi, van Oirschot et al. 1998) | ptxS1B |
| ptxA4 | AJ245368 | (Mooi, van Oirschot et al. 1998) | ptxS1D |
| ptxA5 | AJ006151 | (Boursaux-Eude, Thiberge et al. 1999) |  |
| ptxA6 | AJ506994 | (Poynten, McIntyre et al. 2004) | ptxS1F |
| ptxA7 | AJ506995 | (Poynten, McIntyre et al. 2004) | ptxS1G |
| ptxA8 | AY879289 | Wang, Zhang, Lei. Unpublished |  |
| ptxA9 | JX100835 | (Shuel, Jamieson et al. 2013) | ptxS1I |
| ptxA10 | KC981247 | This work |  |
| ptxA11 | JX100836 | (Shuel, Jamieson et al. 2013) | ptxS1H |
| ptxP1 | FN252323 | (Mooi, van Loo et al. 2009) |  |
| ptxP2 | FN252322 | (Mooi, van Loo et al. 2009) |  |
| ptxP3 | FN252324 | (Mooi, van Loo et al. 2009) |  |
| ptxP4 | FN252325 | (Mooi, van Loo et al. 2009) |  |
| ptxP5 | FN252326 | (Mooi, van Loo et al. 2009) |  |
| ptxP6 | FN252327 | (Mooi, van Loo et al. 2009) |  |
| ptxP7 | FN252328 | (Mooi, van Loo et al. 2009) |  |
| ptxP8 | FN252329 | (Mooi, van Loo et al. 2009) |  |
| ptxP9 | FN252330 | (Mooi, van Loo et al. 2009) |  |
| ptxP10 | FN252331 | (Mooi, van Loo et al. 2009) |  |
| ptxP11 | FN252332 | (Mooi, van Loo et al. 2009) |  |
| ptxP12 | FJ980276 | (Advani, Van der Heide et al. 2009) |  |
| ptxP14 | FR854395 | Hegerle, Paris, Brun, Dore, Njamkepo, Guillot, Guiso. Unpublished |  |
| ptxP15 | HM440343 | (Advani, Gustafsson et al. 2011) |  |
| ptxP16 | HM440344 | (Advani, Gustafsson et al. 2011) |  |
| ptxP17 | HM440341 | (Advani, Gustafsson et al. 2011) |  |
| ptxP18 | HM440342 | (Advani, Gustafsson et al. 2011) |  |
| ptxP19 | JQ029160 | (Lam, Octavia et al. 2012) |  |

Advani, A., L. Gustafsson, et al. (2011). "Appearance of Fim3 and ptxP3-Bordetella pertussis strains, in two regions of Sweden with different vaccination programs." Vaccine **29**(18): 3438-3442.

Advani, A., H. G. Van der Heide, et al. (2009). "Analysis of Swedish Bordetella pertussis isolates with three typing methods: characterization of an epidemic lineage." J Microbiol Methods **78**(3): 297-301.

Boursaux-Eude, C., S. Thiberge, et al. (1999). "Intranasal murine model of Bordetella pertussis infection: II. Sequence variation and protection induced by a tricomponent acellular vaccine." Vaccine **17**(20-21): 2651-2660.

Han, H. J., K. Kamachi, et al. (2008). "Antigenic variation in Bordetella pertussis isolates recovered from adults and children in Japan." Vaccine **26**(12): 1530-1534.

Lam, C., S. Octavia, et al. (2012). "Selection and emergence of pertussis toxin promoter ptxP3 allele in the evolution of Bordetella pertussis." Infect Genet Evol **12**(2): 492-495.

Mastrantonio, P., P. Spigaglia, et al. (1999). "Antigenic variants in Bordetella pertussis strains isolated from vaccinated and unvaccinated children." Microbiology **145 ( Pt 8)**: 2069-2075.

Mooi, F. R., Q. He, et al. (1999). "Variation in the Bordetella pertussis virulence factors pertussis toxin and pertactin in vaccine strains and clinical isolates in Finland." Infect Immun **67**(6): 3133-3134.

Mooi, F. R., I. H. van Loo, et al. (2009). "Bordetella pertussis strains with increased toxin production associated with pertussis resurgence." Emerg Infect Dis **15**(8): 1206-1213.

Mooi, F. R., H. van Oirschot, et al. (1998). "Polymorphism in the Bordetella pertussis virulence factors P.69/pertactin and pertussis toxin in The Netherlands: temporal trends and evidence for vaccine-driven evolution." Infect Immun **66**(2): 670-675.

Poynten, M., P. B. McIntyre, et al. (2004). "Temporal trends in circulating Bordetella pertussis strains in Australia." Epidemiol Infect **132**(2): 185-193.

Schmidtke, A. J., K. O. Boney, et al. (2012). "Population Diversity among Bordetella pertussis Isolates, United States, 1935-2009." Emerg Infect Dis **18**(8): 1248-1255.

Shuel, M., F. B. Jamieson, et al. (2013). "Genetic analysis of Bordetella pertussis in Ontario, Canada reveals one predominant clone." Int J Infect Dis.

Tsang, R. S., A. K. Lau, et al. (2004). "Polymorphisms of the fimbria fim3 gene of Bordetella pertussis strains isolated in Canada." J Clin Microbiol **42**(11): 5364-5367.

van Gent, M., M. J. Bart, et al. (2012). "Small mutations in Bordetella pertussis are associated with selective sweeps." PLoS One **7**(9): e46407.

van Loo, I. H., K. J. Heuvelman, et al. (2002). "Multilocus sequence typing of Bordetella pertussis based on surface protein genes." J Clin Microbiol **40**(6): 1994-2001.
