# Supplementary material for: Global Population Structure and Evolution of Bordetella pertussis and Their Relationship with Vaccination
Source: mBio. 2014 Apr 22;5(2):e01074-14. doi: 10.1128/mBio.01074-14 (PMC3994516; doi:10.1128/mBio.01074-14)
Supplement: Text S2 — Polymorphisms in the bvgA and fhaB promoter region. Download [file mbo002141804sd9.pdf]

1968400 1968450

CATGATAAGAAGAATATGCTTTGAGTTTCGTGGCGAGAAAAAAATCCTGCAACGCGTAATGTCATTGATGCGTCAAAGGAATGGCTGCGAAATTTCACA

◄**BvgA** P1◄

1968500 1968550

TTCTCCTATACGTCAGCAAGCAACGATACGGGCATGAGTGCTGCTAAACAGTATCCTATACCAAAATAGGAAAAGTCTGAATTCCTGCGCTGGCACCCGC

P2◄ **C** -10 **T** -35 **C T**

1968600 1968650

GGCGGGCCGGGGAGCGGGTTGTCGGCGCACGCCTATACGTGCCGGACAGGGTTTGATGGTTTGACTAAGAAATTTCCTACAAGTCTTGTATAAATATCCA

A **A**

T

1968700

-35 -10 ►P*fhaB* 1968750

TTGATGGACGGGATCATTACTGACTGACGAAGTGCTGAGGTTTATCCAGACTATGGCACTGGATTTCAAAACCTAAAACGAGCAGGCCGATAACGGATTC

G **G** **G** **A** **A**

TGCCGATTACTTCACTTCGCTGGTCGGAAT**ATG** ►**FhaB**

Supplemental Text S2. Polymorphisms in the *bvgA* and *fhaB* promoter region. The *bvgA* and *fhaB* are faced back-to-back and transcribed to the left and right in this figure, respectively. The *bvgA* and *fhaB* initiations codons are in bold. Start and direction of transcription are indicated by arrowheads marked with P1, P2 and P*fhaB* (Scarlato et al 1990). The locations of the -35 and -10 elements for *bvgA* and *fhaB* are indicated by blue and grey boxes, respectively. Both the *bvgA* and the *fhaB* gene are positively regulated by BvgA, and the BvgA binding sites are denoted by green boxes (Decker et al 2011). The identified SNPs are indicated in red below the promoter region sequence. Numbering is based on the Tohama I sequence (Parkhill et al., 2003).
