# Supplementary material for: Global Population Structure and Evolution of Bordetella pertussis and Their Relationship with Vaccination
Source: mBio. 2014 Apr 22;5(2):e01074-14. doi: 10.1128/mBio.01074-14 (PMC3994516; doi:10.1128/mBio.01074-14)
Supplement: Figure S1 — Maximum likelihood phylogenetic tree of all isolates included in this study (see Table S1). (A) The complete tree of all isolates rooted on a ST15 B. bronchiseptica isolate, strain MO149. The branch leading to MO149 has been shortened to allow the relationships within B. pertussis to be seen. (B) A zoomed view of the maximum likelihood tree of lineage II to allow the relationships in this lineage to be more clearly seen. Scale bars indicate numbers of SNPs. Download [file mbo002141804sd3.pdf]

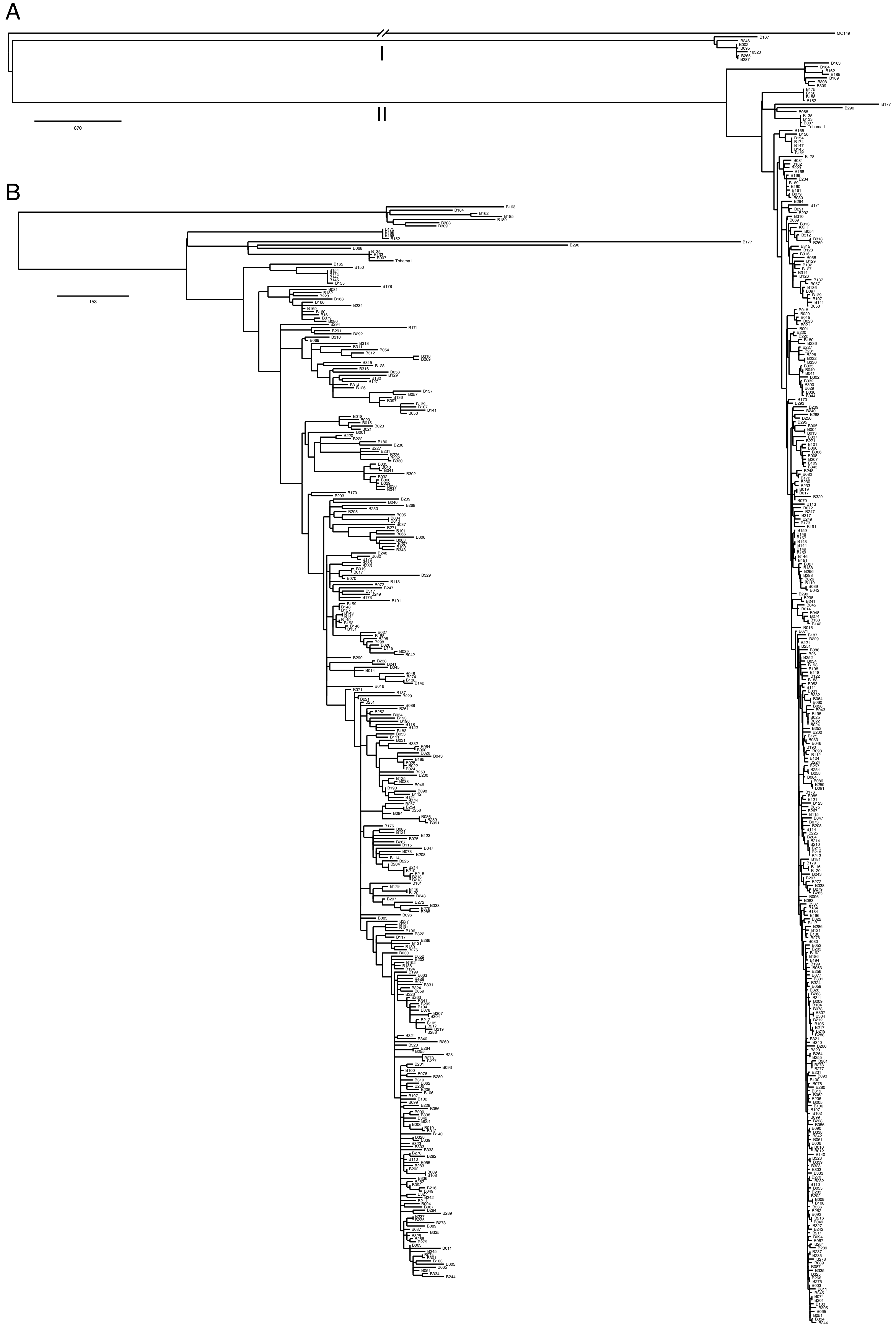
A
I
II
870
B
153
